# Ethnobotanical study of plants used in management of livestock health problems by Afar people of Ada’ar District, Afar Regional State, Ethiopia

**DOI:** 10.1186/1746-4269-9-8

**Published:** 2013-01-23

**Authors:** Mirutse Giday, Tilahun Teklehaymanot

**Affiliations:** 1 Aklilu Lemma Institute of Pathobiology, Addis Ababa University, P.O. Box 1176, Addis Ababa, Ethiopia

**Keywords:** Medicinal plants, Ethnoveterinary practices, Afar, Ada’ar District, Ethiopia

## Abstract

**Background:**

The great majority of the Afar people of Ethiopia are pastoralists, highly dependent on livestock and livestock products. Livestock productivity is, however, frequently affected by different diseases. Although many districts in the Region have veterinary clinics, they lack basic facilities. As a result, the Afar people are still dependent on local materials, mainly plants, and traditional knowledge to manage livestock health problems. However, there is a serious threat to such local resources mainly due to recurrent drought and influence of modernization. Hence there is a need for proper documentation and evaluation of the existing ethnoveterinary knowledge in the Region. This study was aimed at documenting and analysing ethnoveterinary knowledge of people in Ada’ar District of the Afar Region associated with the use of plants.

**Methods:**

The study involved interviewing selected knowledgeable Afar people in Ada’ar District on the use of plants to manage livestock ailments. Fidelity Level (FL) values were calculated for the reported medicinal plant to estimate their healing potentials. Specimens of reported medicinal plant were collected, identified and deposited at the National Herbarium, Addis Ababa University.

**Results:**

The study revealed 49 medicinal plants as being used by the Afar people of Ada’ar District for the treatment of various livestock ailments, the majority of which (67.3%) were shrubs. Highest number of medicinal plants was used to treat blackleg, contagious caprine pleuropneumonia (CCPP), sudden sickness and pneumonia. Leaf was the most frequently sought plant part, accounting for 47% of the reported plants. All the medicnal plants used in the District were uncultivated ones growing in semi-disturbed and disturbed habitats as remnant plants and weeds. *Cissus quadrangularis* and *Solanum incanum* were the plants scoring the highest fidelity level values for their use to treat blackleg and respiratory tract problems, respectively.

**Conclusion:**

The study revealed that there is still rich knowledge of ethnoveterinary medicine in Ada’ar District. There was no habit of cultivating medicinal plants by people in the study area. Efforts, should, therefore, be made to protect these medicinal plants from further depletion, especially those that are scarcely availabale. Better attention should be given to medicinal plants with the highest fidelity level values as such values could indicate potencies of the plants.

## Background

Ethiopia is among the countries in Africa with the highest livestock populations [1]. Livestock production is an integral part of the Ethiopian agriculture and shares about 40% of the total agricultural output [2]. Although the country is rich in its livestock population, it is among the countries in the world with the lowest unit output. The poor health condition of its livestock has partially been responsible for the low productivity.

Modern livestock health care is still at its lowest stage in the country due to lack of adequate clinics, veterinarians and supply of drugs. Besides, most modern drugs are expensive and, as a result, not affordable by the majority of Ethiopian farmers and pastoralists. As a result, people rely on their traditional knowledge, practices and locally available materials (mainly plants) in the management of diseases of their domestic animals. Ethnoveterinary practice in the country is, however, being affected due to acculturation and depletion of plants as a result of environmental degradation, deforestation and over exploitation of the medicinal plants themselves. However, very little of the ethnoveternary knowledge in Ethiopia in relation to the use of medicinal plants is so far properly documented and analysed [3-8].

The Afar people of Ethiopia reside in the Afar Regional State. The great majority of them are pastoralists and, as a result, highly dependent on livestock and livestock products. Livestock productivity is, however, frequently affected by different diseases. They, therefore, give high priority to the well being of their domestic animals. As access to modern veterinary facilities in the Afar Region is very limited, the people are still expected to largely rely on their traditional knowledge in the management of livestock health problems.

To the author’s knowledge, there is only one proper ethnobotanical study conducted in the Afar region [9]. The current study tried to document and analyse ethnoveterinary knowledge and practices of the pastoral Afar people residing in Ada’ar District of the Afar Region with emphasis on medicinal plants. Ada’ar is among the districts in the Region with the highest populations of cattle, goats and sheep.

## Materials and methods

### The Afar Region and people

The Afar people reside in the Afar Regional State, north-eastern Ethiopia. Afar is one of the major pastoral regions in Ethiopia. It shares borders with Eritrea in the north, Djibouti and Somali Region in the east, Tigray and Amhara regions in the west and Oromiya Region in the south. Semera is the administrative town of the Region and is located at about 600 km northeast of Addis Ababa. The Afar Region is divided into five zones, 29 districts (weredas) and 355 kebeles [10]. Kebele is the smallest administrative unit in Ethiopia. Altitude in the Region ranges from 1500 m a.s.l. to 120 m below sea level [10]. The Region receives bimodal rainfall with a precipitation of 150–500 mm per annum and temperature varies from 20 to 48°C [10].

Based on the 1994 national census, the population of the Afar people of Ethiopia was projected to be 1.3 million in 2004, of which 56% are males and 44% females [11]. Afar economy is highly dependent on livestock and livestock products. The livestock population of the Afar Region is estimated to be about 4 million [10]. Cattle, sheep, goats, camels and donkeys are commonly raised. Livestock productivity is, however, affected by different diseases that occur in the Region [12]. Although the majority of districts in the region have veterinary clinics, they lack basic facilities such as electricity, water and clinical and diagnostic equipment and most of them frequently face shortage of drugs [10]. Afar people residing in districts adjacent to the neighbouring highlands of the Tigray and Amhara regions cultivate sorghum, maize, teff and cotton [10].

### The study district

**Figure 1:**
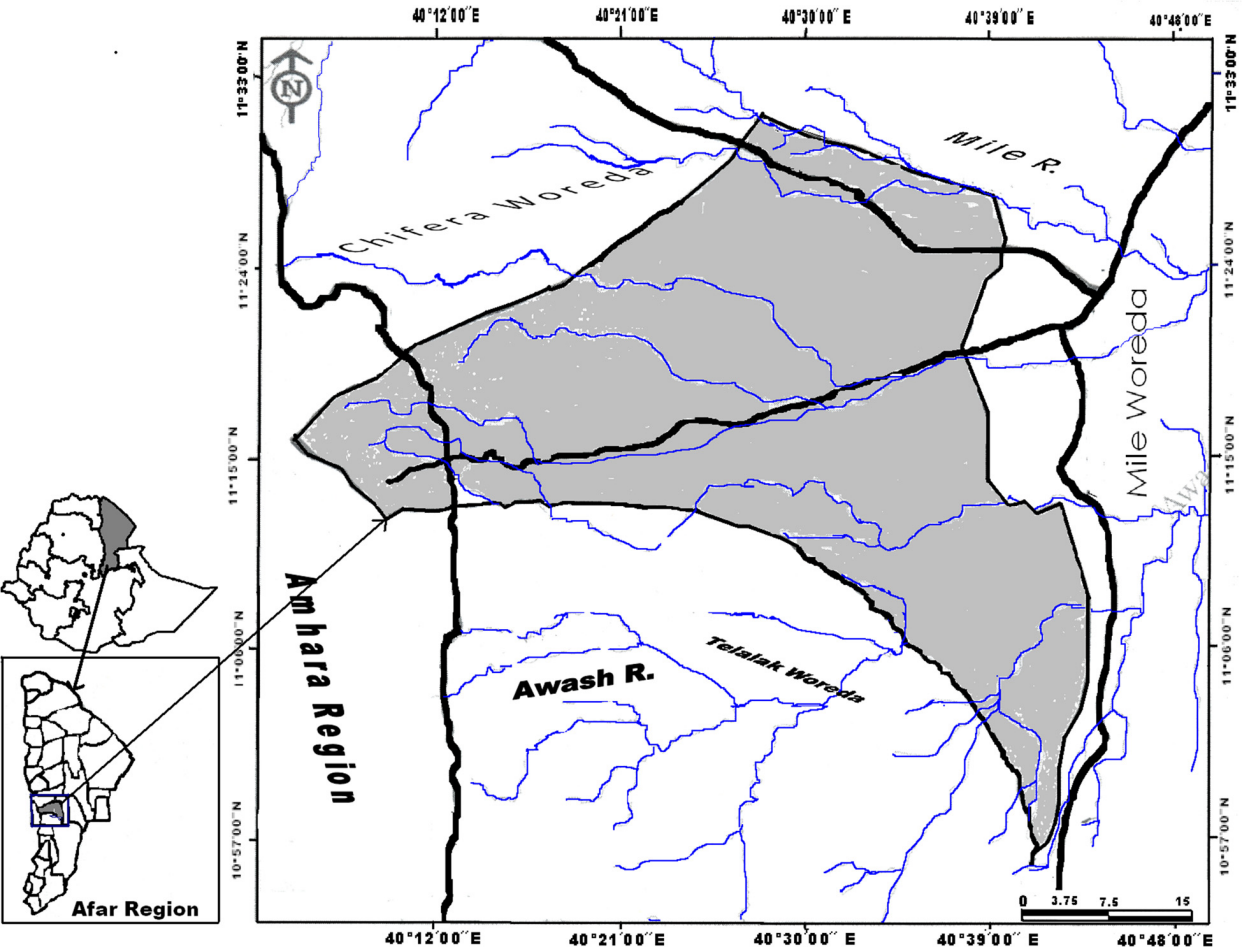
Map of Ada’ar District (Source: GIS Team of Afar National Regional State, BoFED, May 2009).

The current study was conducted in Ada’ar District belonging to Zone 1 of the Afar Region of Ethiopia (Figure 1). The District is bordered by Mile (east), Chifra (northwest) and Dewe (southeast) districts of the Afar Region and Bati (west) and Telalak (southwest) districts of the Amhara Region. The District is administratively divided into 13 kebeles. According to Ada’ar District Administration, the total human population of the District is 59,637. According to Ada’ar District Pastoral, Agriculture and Rural Development Office, livestock population of the District in 2009 was estimated to be 683,977, of which cattle had the highest population (166,964), followed by sheep (164,857) and goat (329,714). The same Office reported that anthrax, blackleg and pastereulosis were among the most prevalent livestock ailments in the District. There are three veterinary health posts and two private drug shops in the District.

### Selection of informants

For the interview, a total of 21 knowledgeable Afar informants (20 males and 1 female) from eight kebeles, namely Burka, Neile-Askala, Siylu-Waky, Ada’ar, Ledi, Woantu-Fursa, Eluwuha and Jeldi were involved. All the informants were Muslims between the ages of 24 and 68 years. The informants were identified with help of Ada’ar Administration Office and elders nominated by the same office.

### Data collection method and type of data collected

Ethnobotanical data were gathered mainly through individual interviews conducted with the knowledgeable informants during five trips made to the study area between August 2008 and April 2011. For this purpose, a semi-structured interview guide was prepared in English beforehand. Interviews with informants were conducted by the researchers with the help of Afar translators. During interview, data regarding the kinds of livestock ailments treated or prevented and the types of medicinal plants used (including their local names), the plant parts used, modes of remedy preparations, route of administration and dosages were collected. Besides, information in relation to local trade, cultivation practices, habitat, abundance and threats of claimed medicinal plants was gathered. Specimens of the reported medicinal plant were collected, dried and identified by their scientific name, and vouchers were deposited at the National Herbarium, Addis Ababa University.

### Data analyzing methods

Microsoft Excel spreadsheet software was employed for organising and analysis ethnobotanical data. Relative importance (RI) of each cited medicinal plant was calculated using a method by Bennett and Prance [13]. RI is calculated using the formula RI = NP + NCS, where NP is obtained by dividing the number of properties (reported specific ailments) attributed to a species divided by the total number of properties attributed to the most versatile species (species with the highest number of properties). NCS is the number of body systems (ailment categories) treated by a given species divided by the total number of body systems treated by the most versatile species. Species with RI value of 2.0 (the highest possible value) are the ones with the highest diversity of medicinal application.

Relative healing potential of each medicinal plant reported was estimated using an index called fidelity level (FL) based on the proportion of informants who agreed on the use of a given medicinal plant against a given major ailment category. The formula for FL is given as FL = I_p_/I_u_ × 100, where I_p_ is the number of informants who independently indicated the use of a species for the same major ailment and I_u_ the total number of informants who mentioned the plant for any major ailment [14]. According to Trotter and Logan [15], plants which are used in some repetitive fashion are more likely to be biologically active.

## Results and discussion

### Medicinal plants reported and ailments treated

A total of 49 medicinal plants were reported to have been used by the Afar people of Ada’ar District for the treatment of different livestock ailments, of which 39 have been identified to a species level (Table 1). Four plants have been identified to genus level and two to a family level. Four plants, for which voucher specimens have not been collected, are only known by their local names. However, attempt has been made to determine the habit of the uncollected plants based on descriptions given by informants. Despite the fact that the study district is an arid land with poor plant cover, a good number of medicinal plants (49 species) are being used in the study area to treat various livestock ailments. This could demonstrate the high dependence of the Afar people in the District on ethnoveterinary service as it is cheaper and easily accessible as compared to modern veterinary service. According to Ada’ar District Pastoral, Agriculture and Rural Development Office, there are only three veterinary health posts and two private drug shops in the whole District. Similar study conducted in Tigray Region of Ethiopia revealed the usage of 44 and 60 medicinal plants in Ofla and Raya-Azebo districts, respectively, for the treatment of different livestock diseases [7]. Ethnoveterinary studies conducted in Dabat District of the Amhara Region [16] and Chiro District of the Oromia Region in Ethiopia [17] uncovered the use of 18 medicinal plants in each district. A recent report [18] indicated the use of 24 medicinal plants by people in Medebay-Zana District of Tigray Region for ethnoveterinary purpose.

**Table 1:** Medicinal plants of the Afar people in Ada’ar District

***Scientific name***	***Family***	***Local name***	***Habit***	***Part used***	***Local disease name***	***English disease name***	***Animal treated***	***Administration route***	***Voucher No.***
*Acacia nilotica* (L.) Willd. ex Del.	Fabaceae	kenselto	tree	fruit	gublo	CCP^1^P	goat	oral	MG-TT-11
					uruga	diarrhea	Goat, sheep	oral, nasal	
*Acacia oerfota* (Forssk.) Schweinf.	Fabaceae	goronto	shrub	bark	geno	sudden sickness	cattle	oral, nasal	MG-TT-14, MG-TT-5
*Acalypha fruticosa* Forssk.	Euphorbiaceae	subahi	shrub	leaf	indahi	CCPP	sheep	oral	MG-TT-12
*Acalypha indica* L.	Euphorbiaceae	baro berbere	herb	whole part	geno	anthrax	cattle, camel	oral	MG-TT-38
				leaf	ladore	blackleg	cattle	oral	
*Aerva javanica* (Burm.f) Schultes	Amaranthaceae	alyaito	shrub	root	intibiaki	ophthalmic infection	goat	ophthalmic	MG-TT-10
*Aloe trichosantha* Berger	Aloaceae	ureita	shrub	leaf	geno	anthrax	goat	oral, nasal	MG-TT-15
				leaf	gublo	CCPP	goat	oral, nasal	
				leaf	gublo	CBPP^2^	cattle	oral, nasal	
*Argemone mexicana* L.	Solanaceae	baro bangi	herb	leaf	abeb	FMD^3^	cattle	oral	MG-TT-45
*Balanites aegyptiaca* (L.) Del.	Balanitaceae	uda	tree	root	finoita	bloat	animals	oral	MG-TT-54
				root	geno	anthrax	cattle, goat, camel	oral	
				root	geno	colic	cattle	oral, nasal	
				above ground	geno	sudden sickness	goat, cattle	oral, nasal	
				root	geremole	trypanosomiasis	camel	oral, nasal	
				root	indahi	CCPP	goat	oral, nasal	MG-TT-19
				root	sole	diarrhoea	cattle	oral, nasal	MG-TT-2
*Balanites rotundifolia* (van Tieghem) Blatter	Balanitaceae	uda	shrub	root bark	kahiw	pneumonia	camel	nasal	MG-TT-49
*Barleria acanthoides* Vahl	Acanthaceae	ganselto	shrub	root	harayiti	blackleg	cattle	oral, nasal, auricular, ophthalmic	MG-TT-47
*Boscia coriacea* Pax	Capparidaceae	urma	shrub	leaf	abeli	babesia	cattle	nasal	MG-TT-24
				leaf	geno	anthrax	cattle	nasal	
				leaf	ladore	blackleg	cattle	oral, nasal	MG-TT-1, MG-TT-22
*Boscia senegalensis* Lam. ex Poiret	Capparidaceae	aitineba	shrub	leaf & fruit	arba	tympanic bloat	cattle	oral	MG-TT-48
*Bourreria orbicularis* (Hutch. & E.A. Bruce) Thulin	Boraginaceae	ulageita	shrub	leaf, bark	dale	wound	animals	local on wound, oral	MG-TT-16
				leaf & bark	harayita	blackleg	cattle	oral, nasal	
*Cadaba farinosa* Forssk.	Capparidaceae	dinibayto	shrub	root	lahi biyak	pastereulosis	cattle	oral, nasal, auricular	MG-TT-29
*Cadaba glandulosa* Forssk.	Capparidaceae	udodoita	shrub	leaf	geno	pneumonia	cattle	oral, nasal	MG-TT-30
				leaf	uruga	diarrhoea	cattle	oral, nasal	
*Cadaba rotundifolia* Forssk.	Capparidaceae	angelita	shrub	leaf	ladore	blackleg	cattle	oral, nasal	MG-TT-07, MG-TT-25
*Calotropis procera* (Ait.) Aitf.	Apocynaceae	gela'eto	shrub	branch, seed	abel	babesia	goat, sheep	nasal, auricular, oral	MG-TT-33
				latex	aray mude	blackleg	cattle	local on legs	
				root	geno	colic	cattle	oral, nasal	
				latex	-	prophylaxis for different diseases	cattle	local on skin	
*Caralluma* sp	Asclepiadaceae	uramo	herb	leaf, stem, whole part, branch	ladore, haraimude	blackleg	cattle	oral, nasal, auricular	MG-TT-17
				above ground	lahi biyak	pastereulosis	cattle	oral, nasal, auricular	
				flower	gublo	CBPP	cattle	oral, nasal	
*Cissus quadrangularis* L.	Vitaceae	Musriga, yaey’eto	climber	leaf, above ground, stem	Harayiti, ladore	blackleg	cattle	oral, nasal, auricular	MG-TT-9, MG-TT-26
*Citrullus colocynthis* (L.) Schrad.	Cucurbitaceae	dearteba	trailing herb	fruit	kehu	pneumonia	camel	oral, nasal, auricular	
				root & leaf	sengite	CBPP	cattle, goat	oral & nasal	MG-TT-8
*Cocculus pendulus* (J.R. & G. Forst) Diels	Menspermaceae	hayofto	climber	root	finoita	bloat	animals	oral	MG-TT-50
*Commicarpus helenae* (J.A. Schultes) Meikle	Nyctaginaceae	kerbeti	herb	whole part	abeli	babesia	goat	nasal, auricular, oral	MG-TT-36
*Cordia* sp.	Boraginaceae	hulten taya'e	climber	above ground	harayita	blackleg	cattle	oral, nasal	MG-TT-21
*Datura stramonium* L.	Solanaceae	gali dimak	herb	fruit, leaf	andelityo	nerve problem	camel	oral	MG-TT-51
*Dobera glabra* (Forssk.) Pair.	Salvadoraceae	garsa, garsaito	shrub	leaf	geno	sudden sickness	cattle, goat	oral, nasal	MG-TT-20
				leaf	kilem	tick infestation	cattle, goat, camel, sheep	oral, local on skin	MG-TT-3
				leaf	ladore	blackleg	cattle	nasal, auricular	
				leaf	geno	bloat	cattle	oral	
*Euphorbia* sp.	Euphorbiaceae	engda'eto	shrub	whole part	sura'ito	CCPP	goat, sheep	oral, nasal	MG-TT-6, MG-TT-52
				whole part, root	uruga, kehu	diarrhoea	sheep, goat	oral, nasal	
				stem	sengite	CBPP	cattle, goat	oral, nasal	
*Grewia villosa* Willd.	Tiliaceae	habeleyta	shrub	bark	alhe	delayed placenta	camel	oral	MG-TT-32
*Heliotropium longiflorum* (ADC. in DC.) Jaub. & Spach.	Boraginaceae	am'ada		leaf	indahi	CCPP	sheep	oral	MG-TT-34
*Justicia schimperiana* (Hochst. ex Nees) T. Anders.	Acanthaceae	werabikela	shrub	leaf	tele	wound	animals	local on wound	MG-TT-39
				leaf	-	snake bite	animals	oral	
*Kanahia laniflora* (Forssk.) R. Br.	Asclepiadaceae	we'a amhala	shrub	leaf	geno	sudden sickness	cattle, goat	oral, nasal	MG-TT-27
*Kleinia squarrosa* Cufod.	Asteraceae	bisilto	shrub	stem	endahi	diarrhoea	goat	oral	MG-TT-42
*Lantana camara* L.	Verbenaceae	dat'hara	shrub	leaf	abeb	FMD	cattle	oral	MG-TT-44
*Monadenium* sp.	Euphorbiaceae	dargudi	shrub	whole part	haraiti	blackleg	cattle	oral, nasal, auricular	MG-TT-43
*Pergularia tomentosa* L.	Asclepiadaceae	ageraboya	shrub	exudate	abeli	babesia	goat	oral	MG-TT-41
*Salvadora persica* L.	Salvadoraceae	ada'ito	shrub	leaf	dale	wound	cattle, camel, goat	local on skin	MG-TT-18
				leaf	geno	sudden sickness	cattle, goat	oral, nasal	MG-TT-4, MG-TT-18
				leaf, above ground	ladore	blackleg	cattle	oral, nasal, auricular	MG-TT-18
*Seddera bagshawei* Rendle	Convolvulaceae	bekil tefere	shrub	whole part	finoita	bloat	cattle, goat, sheep, camel	oral	MG-TT-46
*Senna alexandrina* Mill.	Fabaceae	airogit, senoyta meka	shrub	leaf	ladore	blackleg	cattle	oral	MG-TT-13, MG-TT-35
				leaf	sindera	orf	goat, sheep	oral, nasal	MG-TT-28
				leaf	-	skin infection	donkey	oral	
*Sericocomopsis pallida* (S. Moore) Schinz	Amaranthaceae	admegarto	shrub	root	gublo	CBPP	cattle	oral, local on skin	MG-TT-53
*Solanum hastifolium* Hochst. ex Dunal in DC.	Solanaceae	askena	shrub	root	abeli	babesia	goat	oral, nasal, auricular	MG-TT-13
*Solanum incanum* L.	Solanaceae	kolodo'ita, wakere ku'us	shrub	fruit	kehu	pneumonia	camel	oral	MG-TT-23
				fruit	goshin, sura'ito	CCPP	goat	nasal	
*Trianthema portulacastrum* L.	Aizoaceae	abure	herb	leaf	antibia	ophthalmic infection	animals	ophthalmic	MG-TT-40
*Withania somnifera* (L.) Dunal	Solanaceae	kokerabito, ubalto	shrub	leaf	shelaitu	listeriosis	cattle	oral, nasal	MG-TT-37
				above ground	ladore	blackleg	cattle	oral, nasal, auricular	
*Ziziphus spina-christi* (L.) Desf.	Rhamnaceae	kusra	shrub	leaf	alhe	delayed placenta	camel	oral	MG-TT-31
-	-	ademudu	shrub	whole part	sindera	orf	sheep, goat	oral, nasal	
-	-	asahada	tree	stem, above ground	kehu	pneumonia	camel	oral, nasal, auricular	
-	Asclepiadaceae	ata'ali	herb	whole part	haraita	blackleg	cattle	nasal	
-	Cucurbitaceae	kuranda geita	climber	root	aray mude	blackleg	cattle	oral, nasal	MG-TT-55
-	-	muyaito	shrub	branch	Indahi, sura’ito	CCPP	goat	oral, nasal	
				branch	ladore	blackleg	cattle	oral, nasal	
-	-	sokoli	shrub	above ground	geno	sudden sickness	cattle, goat	oral, nasal	

^1^CCPP = contagious caprine pleuropneumonia.

^2^CBPP = contagious bovine pleuropneumonia.

^3^FMD = foot-and-mouth disease.

Some of the plants documented during the current study are used elsewhere in Ethiopia to treat same/similar livestock diseases [7,9,19,20]. These include *Acacia nilotica* (used to treat diarrhoea); *Acalypha indica* (used against anthrax), *Aloe trichosantha* (used against anthrax, contagious caprine pleuropneumonia and contagious bovine pleuropneumonia), *Balanites aegyptiaca* (used against anthrax), *Calotropis procera* (used against blackleg) and *Dobera glabra* (used against tick infestation).

The reported medicinal plants belonged to 23 families and 36 genera. Asclepiadaceae and Capparidaceae took the better share of the reported plants (five species each), followed by Euphorbiaceae and Solanaceae (four species each). The families Boraginaceae and Fabaceae had three species each and the families Acanthaceae, Amaranthaceae, Balanitaceae, Cucurbitaceae and Salvadoraceae had two species each. The remaining 12 families had one species each. The fact that Asclepiadaceae and Capparidaceae contributed relatively higher number of medicinal plants might be attribited to better abundance of species in the study area belonging to these families. Kers [21] and Goyder [22] reported that species of the two families are conspicous in drier habitats like that of the study area. The majority (67.3%) of the reported Afar medicinal plants were shrubs and this could be due to higher abundance of shruby species in the area that better adapt to arid conditions as compared to plants of other life forms. Few were herbs (18.4%), climbers (8.2%) and trees (6.1%).

**Figure 2:**
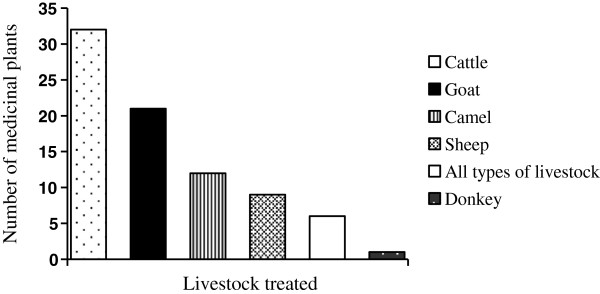
Numbers of medicinal plants used against diffrent livestock ailments.

The plant remedies were prescibed against 23 types of livestock ailments. The majority of the ailments were that of cattle (15), camel (nine) and goat (eight). Six diseases of sheep and one disease of equine were also reported. The higest number of medicinal plants were used to treat blackleg (17 species), contagious caprine pleuropneumonia (eight species), sudden sickness (seven species) and pneumonia (six species) (Table 2). According to unpublished data of Ada’ar District Agriculture and Rural Development Office (ADARD), blackleg and CCPP are among the top five econmically important livestock diseases in the District. The majority of the reported plants were used to treat cattle diseases (32 species), followed by those used against goat (21 species) and camel (12 species) ailments (Figure 2). Based on unpublished data collected in 2009 by ADARD, cattle had the highest population among the livestock in the District, projected to reach 166, 964 heads in 2010.

**Table 2:** List of livestock diseases against which three or more medicinal have been prescribed

**Disease english name**	**Disease local name**	**Number of medicinal plants used in treatment**
Blackleg	ladore	17
Contagious caprine pleuropneumonia	indahi	8
Sudden sickness	geno	7
Pneumonia	kehu	6
Babesia	abel	5
Contagious caprine pleuropneumonia	gublo/sengite	5
Diarrhoea	uruga	5
Anthrax	geno	4
Bloat	finoita	4
External woulnd	dale	4
Tick infestation	kilem	3

Most of the medicinal plants (86%) were used only for curative purpose. However, few (*Boscia coriacea*, *Calotropis procera*, *Dobera glabra*, *Salvadora persica* and *Caralluma* sp.) were used for both curative and prophylactic purposes, and one plant, locally known as ’muyaito’, was only used for prophylactic purpose.

### Parts used and mode of preparation

**Figure 3:**
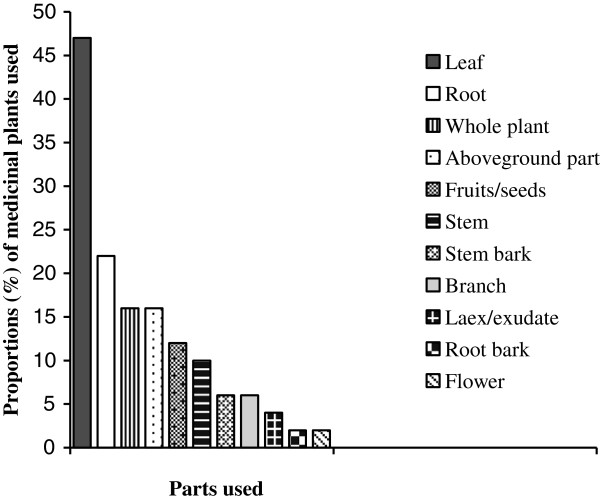
Numbers of medicinal plants harvested for their different parts.

Leaf was the most frequently sought plant part accounting for 47% of the reported medicinal plant species, followed by root (22%), whole plant (16%) and aboveground part (16%) (Figure 3). Similarly in studies conducted elsweher in Ethiopia, leaf was indicated to be the most frequently used plant part to treat livestock ailments [7,18,23]. A study [24] indicated that collection of leaves poses no significant threat to the survival of individual plants as compared to other parts such as underground part, stem, bark and whole plant.

Most of the remedies (86%) were processed and used immediately after collection. Few (*Acacia nilotica*, *Aerva javanica*, *Cadaba glandulosa*, *Caralluma* sp., *Grewia villosa* and *Seddera bagshawei*) were reported to be prepared and adminstered while they were fresh or after drying, and one (asahada) was processed and used only after drying. Remedies of 46 medicinal plants (90%) were prepared in juice form. Remedies of three species (*Acalypha indica*, *Senna alexandrina* and *Caralluma* sp) were prepared ethier in the form of juice or decocton, and that of two (*Kleinia squarrosa* and *Sericocomopsis pallida*) were only prepared in decoction form. There was no pracitice of storing medicinal plants in the study area for future use and this could create shortage of remedies to be used in the District during the dry season when there is scarcity of plants or parts to be harvested.

Preparations of the majority (65%) of remdies invloved single medicinal plant, which is in agreement with the findings of studies conducted elsewhere in Ethiopia [23,25]. However, some (35%) remedies in the current study area were prepared in a concotion form, by mixing two or more medicinal plants, which is similar to the findings of studies carried out eslewhere in the country [8,26]. Many healers in Ethiopia believe that the potency of plant remedies could be enhanced when they are used in concoction form [27].

Water was the most frequently used diluent in the prepartion of remedies accounting for 90% of the medicinal plants and this could be attributed to either its abundance or to the fact that the active principles contained in most plants are water soluble. Remedies from three species (*Boscia coriacea*, *Calotropis procera* and *Solanum incanum*) were prepared with or without the addition of water, and that of *Justicia schimperiana* was prepared without the addtion of a diluent. Remedy from one medicinal plant (*Pergularia tomentosa*) was prepared with the addition of milk as a diluent. Other additives are rarely used. Only butter was reported to be used in the preparation of remedy from *Calotropis procera* against blackleg.

### Route of remedy administration and dosage

Oral was the most frequently used route of remedy administration accounting for 90% the medicinal plants, followed by nasal (61%) and auricular (24%). Some were adminstred locally on the skin or wound (12%) and ophthalmologically (6%). The dominance of oral application of remedies could be related to the fact that most of the reported health problems are affecting internal organs.

Almost all treatments were given on daily basis, the majority of which (83%) were prescribed once a day. Some treatments were given twice (11%), trice (2%) or four times (2%) a day. Most treatments (78%) were reported to be completed within seven days. However, some were (22%) known to be given until cure. The amount of remedy taken at one time varied from disease to disease and from informant to informant, even for the same type of ailment. The dose could range from a few drops to one or more litres. Inconsistency of doses have also been reported in similar studies conducted elsewhere in the country [7,17,28]. All the reported medicinal plants, except one (*Balanites aegyptiaca*), were found not be toxic even taken at higher doses. The root of *Balanites aegyptiaca* could kill animals if given in excess during treatment due its toxic effect.

### Habitat, avialability and marketablity of medicinal plants

All the medicnal plants used in the study District were uncultivated ones growing in semi-disturbed and distrubed habitats as remnant plants and weeds. There was no tradition or practice by local people to cultivate medicinal plants. Plants were harvested and processed only when needs arised. The use of uncultivated plants is a common practice in Ethiopia [29] and this has been creating an addtional pressure on the populations of wild plants besides to enviromental degradation and deforestation.

Most of the plants were commonly available in the study area and could easily be harvested from sites not very far from homesteads. However, the plants *Acacia nilotica*, *Balanites rotundifolia*, *Boscia coriacea*, *Bourreria orbicularis*, *Euphorbia* sp. (engda’eto), *Kanahia laniflora*, *Withania somnifera*, ’ademudu’, ’asahada’, ’muyaito’ and ’sokoli’ were scarcely available in the study area and, therefore, requred longer time to harvest. None of the medicinal plants that were reported from the study area were available for sale in local markets.

### Estimation of medicinal plant healing potential and use-diversity

Fidelity Level (FL), as an estimation of healing potential, was determined for all reported medicinal plants. Accordingly, *Cissus quadrangularis* and *Solanum incanum* were the plants having the highest FL values for their use to treat blackleg and respiratory tract problems, each scoring 100% (Table 3), followed by *Caralluma* sp (86%) and *Euphorbia* sp. (83%). According to Trotter and Logan [15], plants scoring higher informant consensus values are thought to have better potency as compared to plants with less informant consensus values.

**Table 3:** FL values of medicinal plants cited by three or more informants for being used against a given major ailment category

**Plant name**	**Major ailment group**	**I**_**p**_	**I**_**u**_	**FL value (%)**
*Cissus quadrangularis*	Locomotor problems (blackleg)	4	4	100
*Solanum incanum*	Respiratory tract problems (CCPP, pneumonia)	3	3	100
*Caralluma* sp	Locomotor (blackleg)	6	7	86
*Euphorbia* sp.	Respiratory tract problems (CBPP, CCPP, pneumonia)	5	6	83
*Balanites aegyptiaca*	Digestive system problems (bloat, colic, diarrhoea)	3	5	60
	Septicaemic problems (anthrax, sudden sickness, trypanosomiasis)	3	5	60

Estimation of Relative Importance (RI) showed *Balanites aegyptiaca* as having the highest value (1.75), followed by *Calotropis procera* and *Dobera glabra* (Table 4), which could indicate the relative abundance of these plants in the study area. *Balanites aegyptiaca* is used to treat bloat, anthrax, sudden sickness, colic, trypanosomiasis, CCPP and diarrhea. *Calotropis procera* is used against babesia, blackleg and pneumonia, and as prophylaxis against different ailments. *Dobera glabra* is used to cure sudden sickness, tick infestation, blackleg and bloat.

**Table 4:** Relative importance (RI) values for Afar medicinal plants used to treat three or more specific livestock ailments

**Plant name**	**NP**	**NCS**	**RI value**
*Balanites aegyptiaca*	1	0.75	1.75
*Calotropis procera*	0.571	1	1.571429
*Dobera glabra*	0.571	1	1.571429
*Salvadora persica*	0.429	0.75	1.178571
*Euphorbia sp.*	0.571	0.5	1.071429
*Aloe trichosantha*	0.429	0.5	0.928571
*Boscia coriacea*	0.429	0.5	0.928571
*Senna alexandrina*	0.429	0.5	0.928571
*Caralluma sp*	0.429	0.5	0.928571

## Conclusion

In total, 49 medicinal plants were reported to have been used by the Afar people of Ada’ar District in the Afar Region of Ethiopia for the treatment of different livestock ailments. Leaf was the most frequently used plant part in the preparation of remedies. There was no habit of storing medicinal plants in the study area for future use, and as a result people could face shoratge of supply of parts to be used for remedy prepartion in time of need, especially during the dry season. All the medicnal plants used in the study District were uncultivated ones growing in semi-disturbed and distrubed habitats as as remnant plants and weeds. There was no no habit or practice by local people to cultivate medicinal plants. This could pose a threat to those medicinal plants that are scarecely available in the study area. Efforts, should, therefore, be made to save the scarce medicinal plants of the Ada’ar District from futher depletion through in situ and ex situ conservation methods. Special attention should also be given to those medicinal plants that scored the highest fidelity level values as such plants are thought to have better potencies as compared to those having lower fidelity level values.

## Competing interests

The authors declare that they have no competing interests.

## Authors’ contribution

The two authors had significant intellectual contribution towards the design of the study, data collection and analysis and write-up of the manuscript. The authors read and approved the final manuscript.
